# A convolutional attention model classifies copy number variants from whole exome sequencing

**DOI:** 10.1038/s41598-026-44691-2

**Published:** 2026-03-20

**Authors:** Maryem Ouhmouk, Mounia Abik

**Affiliations:** National Higher School For Computer Science and Systems Analysis (ENSIAS), Mohammed V University in Rabat, Rabat, Morocco

**Keywords:** Cancer, Computational biology and bioinformatics, Genetics

## Abstract

Copy number variants are important biomarkers in genetic disease and cancer, yet whole-exome CNV callers often rely on read-depth heuristics that capture limited positional or chromosomal context and generalize poorly across platforms. We present a dual-input convolutional neural network with attention that ingests normalized read depth, genomic coordinates, and chromosome identity. The model was pretrained on ECOLE-labeled 1000 Genomes data and fine-tuned on seven expert-annotated samples. On a held-out test set, the method achieved macro F1 = 0.83 and macro PR-AUC = 0.93. In additional contextual analyses reported in the Supplementary Material, CNN-Att exhibits a sensitivity–precision trade-off consistent with established WES CNV callers. Cross-platform evaluations on HiSeq 4000, NovaSeq 6000, MGISEQ 2000, and BGISEQ 500 yielded overall F1 up to 0.96. Fine-tuning increased deletion and duplication recall at the cost of a higher false positive rate, reflecting an explicit trade-off between sensitivity and precision. The architecture masks padded depth tokens and uses attention to highlight weak depth signals that are characteristic of small or noisy events. These results indicate strong sensitivity and robust performance across sequencing technologies, supporting use in clinical triage, multi-site genomics, and large-scale screening.

**Supplementary Information:**

The online version contains supplementary material available at 10.1038/s41598-026-44691-2.

## Introduction

Copy number variants (CNVs) are genomic segments that are deleted or duplicated relative to a reference genome and represent a pervasive source of human genetic variation^1,2^. Clinically, a lower bound of 1 kb is often applied, although research imposes no strict minimum length^1^. CNVs can be inherited in the germline or arise somatically in cancer; they originate from duplications, deletions, translocations, or inversions and influence common traits, disease risk in cancer and neurodevelopmental disorders, while also shaping population-level evolution^2^. Their detection is therefore central to both rare disease diagnostics and oncology, with recent studies showing that exome-based CNV analysis can identify clinically actionable variants in patients with suspected genetic disorders, complementing single-nucleotide variant (SNV) analysis in routine diagnostic workflows^3^.

Biologically, CNV detection from whole-exome sequencing (WES) is challenging due to uneven probe capture, GC bias, and coverage variability, which produce sparse and noisy depth profiles that obscure true copy number changes. Technically, most established WES CNV callers, such as XHMM^4,5^, CODEX^6^, Control-FREEC^7^, and ExomeDepth^8^ rely on read-depth normalization, principal-component correction, and heuristic thresholds. While these methods can achieve moderate accuracy, they often fail on low-coverage or cross-platform data and lack the capacity to model sequential dependencies and chromosome-level context, leading to reduced sensitivity and generalizability.

Recent deep learning approaches have sought to address these issues by learning feature representations directly from read-depth signals. ECOLE^9^, for instance, uses a transformer architecture with positional encodings and chromosome-specific tokens to integrate positional and chromosomal context into CNV prediction, improving over purely statistical methods. However, transformers are computationally heavy and may require per-platform fine-tuning to retain accuracy.

Here, we present a convolutional neural network with an integrated attention mechanism (CNN-Att) for CNV classification from WES data. The framework encodes chromosome identity via a dense embedding layer, stacks positional and depth features into a fixed-length tensor and applies convolutional filters to capture local depth patterns before attention-based reweighting of the pooled feature representation. This architecture is lightweight and is designed for robust performance across sequencing platforms. Pretrained on WGS-derived CNV labels and fine-tuned on expert-annotated data, CNN-Att achieves strong performance with consistent accuracy across multiple platforms, supporting use in both research and clinical settings.

## Methods

### Data sources and study design

This study utilized CNV-labeled whole-exome sequencing (WES) data from the ECOLE benchmark dataset, which is built from the 1000 Genomes Project. ECOLE provides exon-centered WES coverage profiles together with CNV labels derived from matched WGS-based calls (semi-ground truth), enabling direct comparison of different callers under a unified evaluation protocol. According to Mandiracioglu et al. (2024)^9^, the WES data were generated on Illumina Genome Analyzer II and Illumina HiSeq 2000 using the NimbleGen SeqCap v3 capture kit, while the matched WGS data were generated on NovaSeq. Reads were aligned to the GRCh38 reference genome using BWA-MEM, and semi-ground truth labels were derived by running CNVnator on the matched WGS data; exon windows without deletion/duplication labels are treated as no-call.

From this benchmark (707 samples), we used 300 samples to train CNN-Att and reserved 50 samples as an independent held-out test set. The 50 test samples were never used for early stopping, model selection, or threshold tuning. Sample identifiers for both subsets are listed in Supplementary Dataset 1/Data Availability.

Published performance summaries for XHMM^4,5^, CODEX^6^, Control-FREEC^7^, and ECOLE^9^ from the official ECOLE benchmarking framework are included only as contextual reference (Supplementary Table S3). Because these results were generated on test sets that differ from our held-out 50-sample subset, we do not treat them as a paired per-exon benchmark.

For all evaluations, predictions were mapped to ECOLE exon-centered windows and metrics were computed on the set of windows available for each analysis, as specified above.

To assess robustness across sequencing technologies, we also performed cross-platform testing on NA12878 WES data sequenced on BGISEQ 500, Illumina HiSeq 4000, MGISEQ 2000 and NovaSeq 6000. For this analysis, results were computed on the evaluated exon window subsets defined by the available tool outputs under the ECOLE framework (Table 2).

Additionally, we tested the adaptability of the model to domain-specific expert annotations provided by Chaisson et al.^10^ by employing a curated dataset of seven manually labeled samples. Five were used for fine-tuning, and two were held out for independent assessment. Model performance across all datasets was analyzed using accuracy, precision, recall, and F1-score; specificity; ROC-AUC and PR-AUC; log loss; Cohen’s kappa; and Matthews correlation coefficient (MCC)^11,12^. For clarity, all metrics are computed at the level of individual exon-centered windows, each with a single ground-truth label (no call, deletion, or duplication). Each window yields one prediction and one ground-truth label. We compute a 3 × 3 confusion matrix over windows. For class-wise precision/recall/F1 we use a one-vs-rest definition: for a given class, TP are windows correctly predicted as that class; TN are windows whose ground-truth label is not the class and that are also not predicted as that class. FP include windows predicted as the class when the ground truth is another class (including no-call), and FN include ground-truth windows of the class predicted as another class (including the opposite CNV type). Thus, a DEL predicted as DUP contributes one FN for DEL and one FP for DUP (and vice versa).

All confusion matrices and derived metrics were computed using custom Python scripts built on scikit-learn’s classification utilities.

The key metrics are defined as follows:

$$Accuracy=\frac{TP+TN}{TP+TN+FP+FN}$$

$$Precision=\frac{TP}{TP+FP}$$

$$Specificity=\frac{TN}{TN+FP}$$

$$Sensitivity=Recall=\frac{TP}{TP+FN}$$

$${F}_{1}-score=\frac{2 \times TP}{2 \times TP+FP+FN}=2 \times \left(\frac{Precision \times Recall}{Precision+Recall}\right)$$

### Data preprocessing

Input features included read-depth values, genomic coordinates, and chromosome identifiers. We used preprocessed matrices derived from the ECOLE resources; in these matrices, each exon-centered window is represented by 1002 consecutive genomic bins, forming a fixed-length coverage representation for the exon and its flanking region. For each window, the input comprises 1002 depth tokens (columns 0–1001), followed by the genomic start and end coordinate (columns 1002–1003) and a chromosome identifier (column 1004). We used the 1002 depth tokens as the primary sequential input.

Depth values were min–max normalized to the [0, 1] range (MinMaxScaler). Padded or missing depth tokens were encoded as − 1 and masked during training and inference. Start and end coordinates were independently normalized and then broadcast across the 1002 positions to align with the depth sequence length. The three per-position features (depth, normalized start, normalized end) were stacked into a 3-channel tensor of shape (1002, 3). Chromosome identifiers were one-hot encoded into 24 dimensions (chr1–22, X, Y; MT excluded) and provided as a secondary input.

**Fig. 1:**
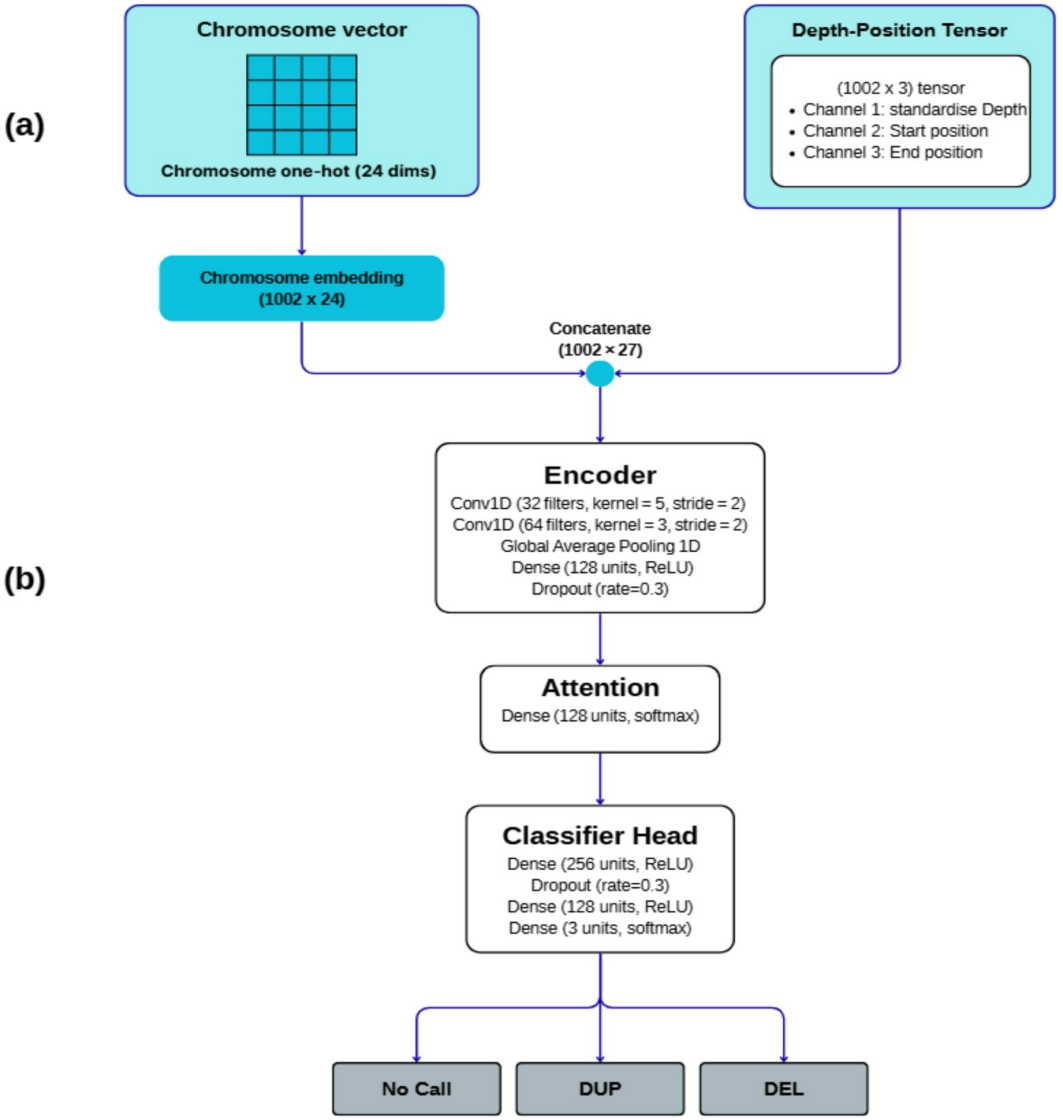
Overview of the CNN-Att architecture for exon centered CNV classification from WES data. (**a**) Input representation. A chromosome one-hot vector (24 dimensions) is embedded and repeated along the sequence to form a chromosome embedding of shape 1002 × 24, which is concatenated with the depth-position tensor (1002 × 3 channels for normalized depth, start position and end position) to produce a joint tensor of shape 1002 × 27. (b) Encoder, attention module and classifier head. The concatenated tensor is processed by two Conv1D layers (32 filters, kernel size 5, stride 2, then 64 filters, kernel size 3, stride 2), followed by global average pooling, a dense layer with 128 ReLU units and dropout 0.3, and a dense attention layer (128 units, softmax). The attention weighted representation is passed to a classifier head with dense layers of 256 and 128 ReLU units and dropout 0.3, and a final 3 unit softmax output that assigns each exon window to one of three classes: no call, duplication (DUP) or deletion (DEL).

### Model architecture and training

The 300-sample training subset contains 435,251 exon windows (before the 70/30 train/validation split); the class distribution is shown in Supplementary Figure S1. Within this training subset, we further divided the windows into training and validation partitions using a 70/30 stratified split, maintaining the original class proportions across no-call, deletion, and duplication labels. To address class imbalance, we applied SMOTE^13^ as implemented in imbalanced-learn, using default hyperparameters (sampling_strategy = “auto”, k_neighbors = 5). In our reference implementation, we fixed the random_state to 42 to ensure reproducibility of the synthetic samples. The training partition was used to optimize model parameters, while the validation partition was used exclusively for early stopping and model selection. The held-out test set (50 samples) was never used during training or early stopping and was reserved strictly for final evaluation.

The classification framework takes two inputs: (i) a primary genomic tensor of shape (1002, 3), where 1002 corresponds to the depth-token sequence length and the three channels correspond to depth, normalized start, and normalized end; and (ii) a one-hot encoded chromosome vector of 24 dimensions. The chromosome vector was passed through a dense embedding layer with 24 units (ReLU activation) and then broadcast across all 1002 positions using a custom RepeatChromosome layer to align dimensions with the primary input.

Feature extraction was performed using two sequential 1D convolutional layers: the first with 32 filters (kernel size = 5, stride = 2), followed by a second with 64 filters (kernel size = 3, stride = 2), both with ReLU activation and same-padding. Instead of max-pooling, global average pooling was applied to the final convolutional output to reduce dimensionality while retaining positional information. The resulting 128-dimensional latent representation was regularized with dropout of 0.3 and passed through a custom softmax-based attention mechanism that reweighted features based on their relative importance in CNV classification.

The attention-weighted output was then passed through two fully connected layers (256 and 128 units, each with ReLU activation and 30% dropout), followed by a final softmax output layer that predicted one of three CNV classes: no-call, deletion, or duplication. The model was optimized using Adam (LR = 0.001) and trained with sparse categorical cross-entropy loss. Early stopping (patience = 10 epochs) and model checkpointing based on validation loss were used to prevent overfitting. All training was performed using TensorFlow on GPU-accelerated hardware (Fig. 1).

The custom RepeatChromosome layer takes the 24-dimensional chromosome embedding vector, repeats it along the sequence axis to produce a tensor of shape (1002, 24), and concatenates this tensor with the primary genomic tensor along the feature dimension.

### Transfer learning procedure

To adapt the model to a limited expert-labeled dataset, we used HG00512, HG00513, HG00731, HG00733, and NA19238 for fine-tuning, and HG00732 and NA19240 for independent evaluation. Starting from the pretrained CNN-Att network, we froze the convolutional encoder and retrained only the classification head, which consisted of two fully connected layers (256 and 128 units, each with ReLU activation and 50% dropout) followed by a softmax output layer. Fine-tuning was performed with a reduced learning rate of 0.0002, using early stopping with a patience of five epochs to prevent overfitting. The checkpoint with the lowest validation loss was saved as the expert-tuned model. Performance on the held-out samples was evaluated using accuracy, precision, recall, F1-score, and ROC-AUC.

## Results and discussion

### Pretrained model performance

The pretrained model exhibited robust classification performance across all CNV categories. Evaluated on 153,435 genomic windows from the 50 held-out test samples, it achieved an overall accuracy of 83%. All metrics in Table 1 were computed by pooling all exon windows across the test samples (window-level evaluation). The area under the receiver operating characteristic curve (ROC-AUC) reached 0.957, reflecting high discriminative capacity between CNV types. Precision–recall analysis further validated this performance, with a mean PR-AUC of 0.925 across classes (Table 1). Class-specific PR-AUC scores were 0.943 for no-call windows (Class 0), 0.938 for deletions (Class 1), and 0.893 for duplications (Class 2).

**Table 1:** Model Performance on the Test Set.

Metric	Class 0 (No-call)	Class 1 (Deletion)	Class 2 (Duplication)	Aggregate
Precision	0.86	0.85	0.79	0.83
Recall	0.85	0.86	0.80	0.83
F1-score	0.85	0.85	0.79	0.83
PR-AUC	0.94	0.94	0.89	0.93
Specificity	0.93	0.93	0.90	0.92

The model maintained balanced performance across classes, with specificity ranging from 0.90 to 0.93 and well-calibrated probabilities (log loss = 0.391) (Supplementary Tables S1–S2). Agreement metrics, including the Matthews correlation coefficient (0.752) and Cohen’s kappa (0.752), indicated strong classification consistency. Although duplications remained the most challenging class, this likely reflects both biological and technical factors. Biologically, duplications often produce smaller relative changes in read depth than deletions, making them harder to detect. Technically, duplications are less frequent in both the training and test data, limiting the model’s exposure to representative patterns.

Because CNN-Att incorporates genomic coordinates and chromosome identity as explicit inputs, we assessed whether performance could be driven primarily by locus-specific priors. We therefore trained a coordinate-only (“locus-prior”) baseline on the same 300-sample training subset used to fit CNN-Att. On a stratified 30% hold-out split of this 300-sample training matrix (130,576 exon windows), the baseline achieved an accuracy of 0.81, a macro F1-score of 0.80, and a CNV recall of 0.77. These values remained below those of CNN-Att on the independent 50-sample test set (macro F1 = 0.83 with higher deletion and duplication recalls), supporting that CNN-Att leverages read-depth patterns in addition to locus-specific priors.

To further address this concern, we stratified true CNV windows (DEL + DUP) in the test set according to how frequently the corresponding exon window was labeled as CNV in the training data. Frequency was defined per exon window as the fraction of the 300 training samples in which that locus was labeled as CNV (DEL or DUP). Four strata were defined: Never (0%), Rare (0–5%), Often (5–50%), and Majority (> 50%). No true CNV windows in the held-out test set fell into the Rare (0–5%) stratum. CNN-Att retained strong recall for CNVs in the Never stratum (*n* = 6,153), with CNV recall = 0.88 (DEL recall = 0.86; DUP recall = 0.64), compared with CNV recall = 0.94 in the Majority stratum (*n* = 30,798). The Often stratum contained relatively few CNV windows (*n* = 120) and should therefore be interpreted cautiously. Overall, recall did not collapse for exon windows never observed as CNV during training, supporting generalization beyond locus-specific priors (Supplementary Table S7). Stratified performance is reported on true CNV windows only, as requested by the reviewer, to specifically assess whether recall decreases for loci not observed as CNV during training.

### Contextual reference to established WES CNV callers

For contextual reference, published performance summaries for XHMM, CODEX, Control-FREEC, and ECOLE under the official ECOLE benchmarking framework are provided in Supplementary Table S3. These values were generated under the ECOLE protocol on test sets that differ from the 50-sample subset used to evaluate CNN-Att in this study and therefore do not constitute a paired per-exon head-to-head comparison on identical samples.

All primary quantitative conclusions in this work are based on the held-out 50-sample evaluation presented in Table 1 and the cross-platform robustness experiments presented in Table 2. A strict paired benchmark that evaluates all tools on identical samples and counts missing predictions as false negatives would require rerunning all baseline callers under identical conditions, which was beyond the scope of the present study.

### Cross-platform generalizability

To evaluate robustness across sequencing technologies, we assessed performance on WES data from HiSeq 4000, MGISEQ 2000, NovaSeq 6000, and BGISEQ 500. Table 2 presents performance metrics across platforms. CNN-Att achieved overall F1-scores of 0.89–0.96 across platforms, while traditional tools showed larger drops, especially for deletions and duplications.

**Table 2:** Cross-Platform Performance Comparison (F1-scores).

Platform	Tool	DEL F1	DUP F1	NC F1	Overall F1
HiSeq 4000	XHMM	0.00	0.00	0.62	0.28
	CODEX	0.20	0.31	0.53	0.39
	**ECOLE**	0.66	0.86	0.83	0.80
	CNN‑Att	**0.90**	**0.88**	**0.90**	**0.89**
MGISEQ 2000	XHMM	0.00	0.00	0.73	0.41
	CODEX	0.14	0.10	0.57	0.37
	ECOLE	0.55	0.72	0.84	0.76
	CNN‑Att	**0.96**	**0.93**	**0.97**	**0.96**
NovaSeq 6000	XHMM	0.00	0.00	0.64	0.30
	CODEX	0.21	0.33	0.59	0.42
	ECOLE	0.60	0.73	0.78	0.73
	CNN‑Att	0.83	0.90	0.93	0.90
BGISEQ 500	XHMM	0.00	0.00	0.75	0.45
	CODEX	0.00	0.06	0.60	0.30
	ECOLE	0.64	0.77	0.87	0.81
	CNN‑Att	**0.90**	**0.95**	**0.97**	**0.94**

**Fig. 2:**
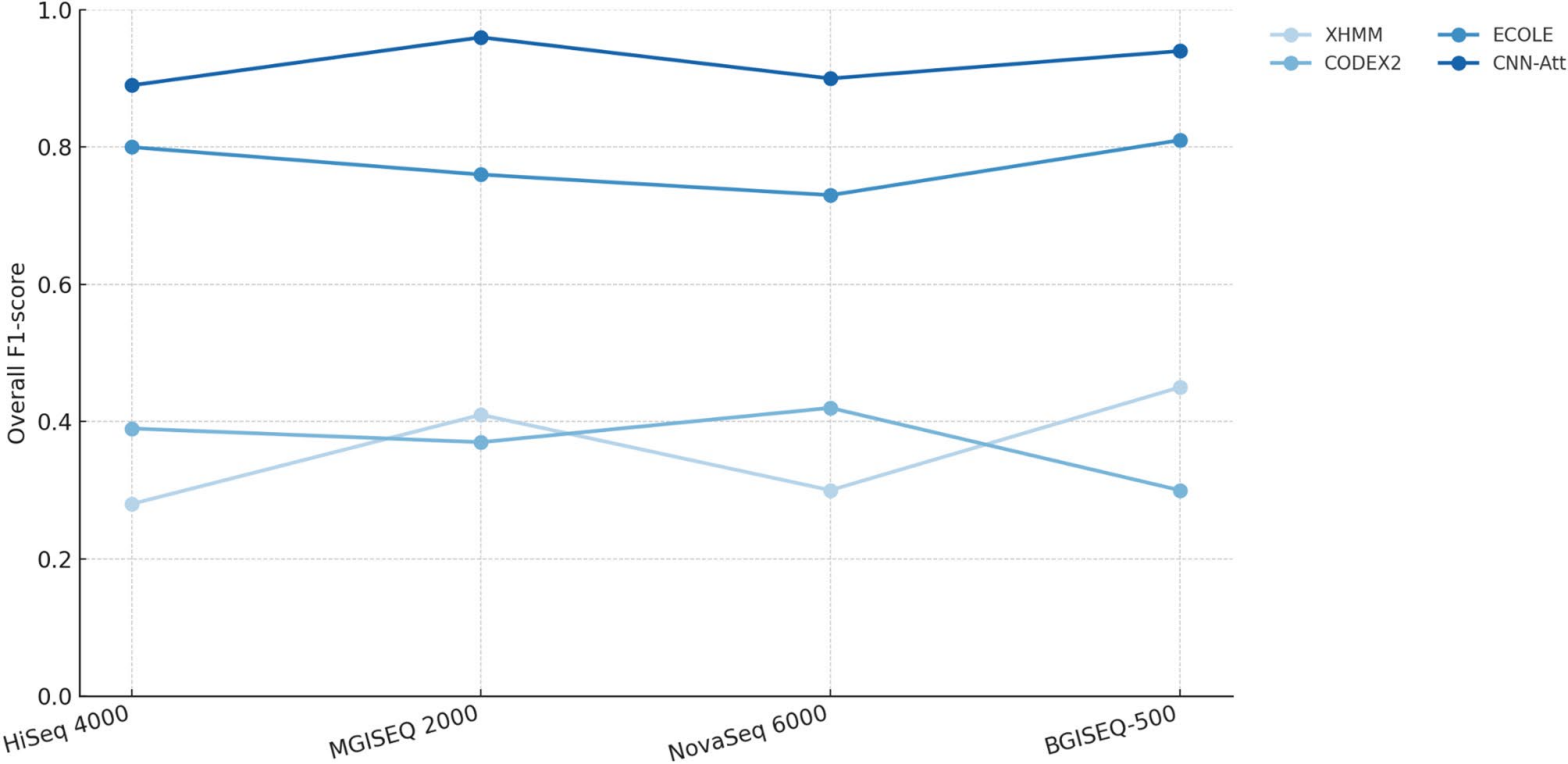
Overall F1-scores across four sequencing platforms (HiSeq 4000, MGISEQ 2000, NovaSeq 6000, and BGISEQ 500) for XHMM, CODEX, ECOLE, and CNN-Att.

For the cross-platform analysis, metrics were computed on exon windows with ECOLE semi-ground-truth labels, using the subset of windows available for each caller/platform under the ECOLE framework. Because the NA12878 cross-platform evaluation uses a different (and typically easier) labeled window subset than the pooled 50-sample test set, the absolute F1-scores in Table 2 are not directly comparable to those in Table 1 and are expected to be higher. The slightly lower overall F1 on NovaSeq compared with MGISEQ (0.90 vs. 0.96) may reflect platform-specific differences in capture chemistry and coverage uniformity that produce more challenging depth profiles for some exons. Overall, CNN-Att showed robust performance across sequencing technologies under the ECOLE-defined evaluated subsets (Fig. 2).

### Transfer learning on expert‑curated data

On sample NA19240, our model achieved an F1-score of 0.66 overall, compared to 0.33 for ECOLE. Deletion and duplication F1-scores increased from 0.02 to 0.00 (ECOLE) to 0.63 and 0.35, respectively. No-call precision also improved from 0.49 to 0.65, with a corresponding increase in F1-score from 0.66 to 0.72 (Supplementary Table S5).

On sample HG00732, the overall F1-score increased from 0.48 (ECOLE) to 0.61. Recall for deletions and duplications improved from 0.03 to 0.06 to 0.38 and 0.58, respectively, though with some reduction in precision (Supplementary Table S6).

These results indicate that, even with limited curated data, fine-tuning can increase sensitivity while keeping precision at levels that are still workable for follow-up validation assays. Because the expert-labeled set includes only seven samples, these findings should be regarded as proof of concept rather than evidence of clinical readiness, and larger curated cohorts will be needed to obtain more robust performance estimates. Future work will extend this analysis to broader expert-labeled datasets and explore strategies such as semi-supervised learning and confidence-based sample selection to improve robustness in low-data settings. Although long-read and whole-genome sequencing offer more detailed structural variant information, their cost and computational demands limit their use in large-scale clinical screening. WES is already widely used as a frontline diagnostic test, and by improving exon-level CNV sensitivity on WES data, CNN-Att can strengthen existing WES-based pipelines and help narrow the gap with WGS or long-read CNV detection, particularly for diagnostic triage and large-cohort studies.

## Conclusion and future work

This study describes a deep learning framework for classifying copy number variants (CNVs) from whole-exome sequencing (WES) data. By encoding genomic depth, segment positions and chromosome identity into a structured tensor and applying a convolutional neural network with attention, the model can learn patterns that are not explicitly modeled in traditional tools. In our experiments on the held-out ECOLE subset, the model achieved high recall while maintaining a balanced overall F1-score, reflecting a sensitivity–precision trade-off comparable to established WES CNV callers.

The model showed balanced performance across classes on the held-out ECOLE subset (macro F1 = 0.83; macro PR-AUC = 0.93), with class-wise recall ranging from 0.80 to 0.86 and precision from 0.79 to 0.86 (Table 1). Cross-platform evaluation on HiSeq, NovaSeq, MGISEQ and BGISEQ datasets indicated good generalizability, with overall F1-scores between 0.89 and 0.96. Fine-tuning on expert-labeled samples further increased sensitivity, at the cost of additional false positives, which may be acceptable in workflows where orthogonal validation is routinely performed and illustrates that the framework can adapt to curated datasets of limited size.

Taken together, these results suggest that CNN-Att may be useful in research and preclinical settings where high recall and cross-platform robustness are important. Higher recall reduces the risk of missed events, while good precision in the no-call class helps to limit unnecessary downstream validation. Its robustness across platforms and ability to incorporate expert input make it a potential framework for future multi-site studies and population-scale genomic projects, and, with further validation, for eventual use in clinical screening pipelines.

In future work, we plan to explore the integration of complementary omics data such as RNA-seq or methylation profiles to improve interpretability and support downstream functional analysis, and to optimize runtime and memory usage for deployment in resource-constrained or real-time environments.

Despite these encouraging results, the framework has several limitations. First, it currently relies exclusively on WES-derived features and does not incorporate transcriptomic or epigenetic information that could enhance biological interpretability. Second, the expert-labeled fine-tuning set is small, which limits the statistical robustness of the transfer learning results^14–16^.

A strict per-exon benchmark that penalizes missing predictions as false negatives would require rerunning all baseline tools on the identical test samples and retaining all benchmark windows. Because CNN-Att was evaluated on a subset of ECOLE samples and baseline tool outputs were generated under the official ECOLE benchmarking framework on different test sets, such a fully paired evaluation was not feasible in the present study. Future work will include direct head-to-head benchmarking on identical sample sets.

Finally, because the model is trained on GRCh38-specific coordinates, it is not directly portable to other reference genomes. Retargeting it to GRCh37 or future builds would require regenerating the input matrices under the new reference and retraining or fine-tuning on labels aligned to that genome build.

## Supplementary Information

Below is the link to the electronic supplementary material.

Supplementary Material 1

## Data Availability

The 1000 Genomes Project WES exon sample names used for training and fine-tuning are available at https://figshare.com/articles/dataset/WES_exon_sample_names/30260350. The labeled ECOLE resources used in this study, including exon-level WES inputs and corresponding labels for the 1000 Genomes Project, Chaisson et al., and NA12878 samples, are available in the official ECOLE repository (https://zenodo.org/record/8202814). The raw whole-exome sequencing (WES) samples from the 1000 Genomes Project can be accessed at https://ftp.1000genomes.ebi.ac.uk/vol1/ftp/data_collections/1000_genomes_project/data/.The full implementation of CNN-Att, including the model definition, training and evaluation scripts, and preprocessing utilities for reconstructing the training and test sets is available in this repository: https://github.com/Ouhmouk-Maryem/CNN-Att.
