# Supplementary material for: A convolutional attention model classifies copy number variants from whole exome sequencing
Source: Sci Rep. 2026 Mar 20;16:14310. doi: 10.1038/s41598-026-44691-2 (PMC13144456; doi:10.1038/s41598-026-44691-2)
Supplement: Supplementary file 1 — Supplementary Material 1 [file 41598_2026_44691_MOESM1_ESM.docx]

### **Pretraining**

**
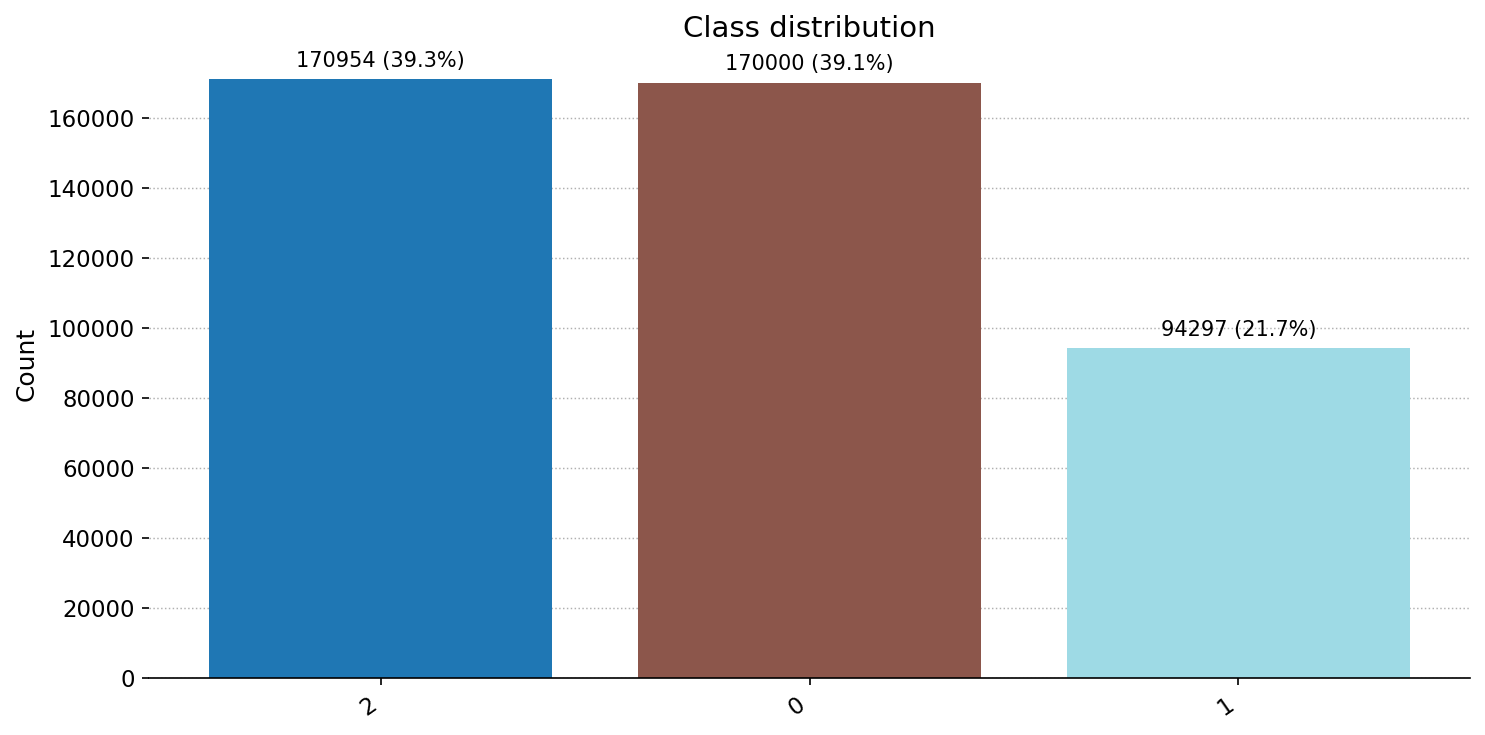
**

***Supplementary Figure S1.*** *Class distribution of the Training dataset*

***Supplementary Table S1.*** *Detailed classification metrics for the pretrained CNN-Att model on the test dataset (153,435 windows)*

| **Class** | **Precision** | **Recall** | **F1‑Score** | **Support** |
| --- | --- | --- | --- | --- |
| **Class 0** | 0.86 | 0.85 | 0.85 | 51,145 |
| **Class 1** | 0.85 | 0.86 | 0.85 | 51,145 |
| **Class 2** | 0.79 | 0.80 | 0.79 | 51,145 |
| **Accuracy** | | | **0.83** | 153,435 |
| **Macro Avg** | 0.83 | 0.83 | 0.83 | 153,435 |
| **Weighted Avg** | 0.83 | 0.83 | 0.83 | 153,435 |

***Supplementary Table S2.*** *Additional performance metrics of CNN-Att model*

| **Metric** | **Value** |
| --- | --- |
| **Test AUC** | 0.9574 |
| **Class 0 PR‑AUC** | 0.9430 |
| **Class 1 PR‑AUC** | 0.9380 |
| **Class 2 PR‑AUC** | 0.8931 |
| **Mean PR‑AUC** | 0.9247 |
| **Matthews Correlation Coefficient (MCC)** | 0.7515 |
| **Cohen’s Kappa** | 0.7515 |
| **Log Loss** | 0.3908 |
| **Sensitivity (Recall) / Specificity** | |
| **• Class 0** | 0.8520 / 0.8578 |
| **• Class 1** | 0.8553 / 0.8537 |
| **• Class 2** | 0.7956 / 0.7918 |
| **Balanced Accuracy** | 0.8343 |


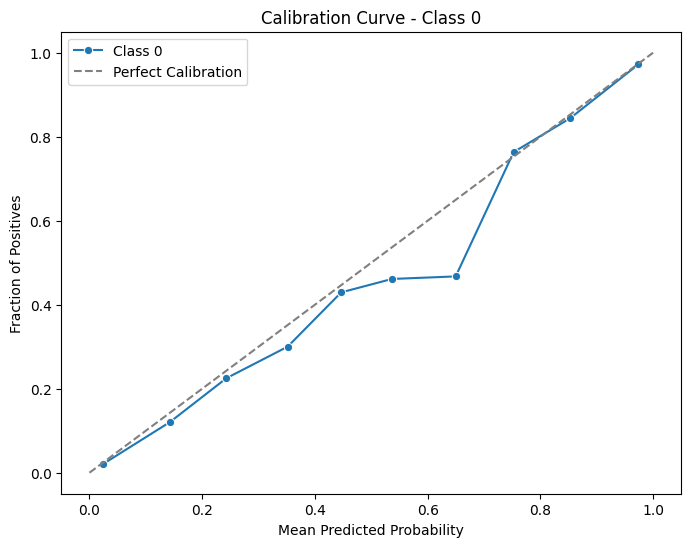


***Supplementary Figure S2.*** *Calibration curve for the No-Call class predictions*


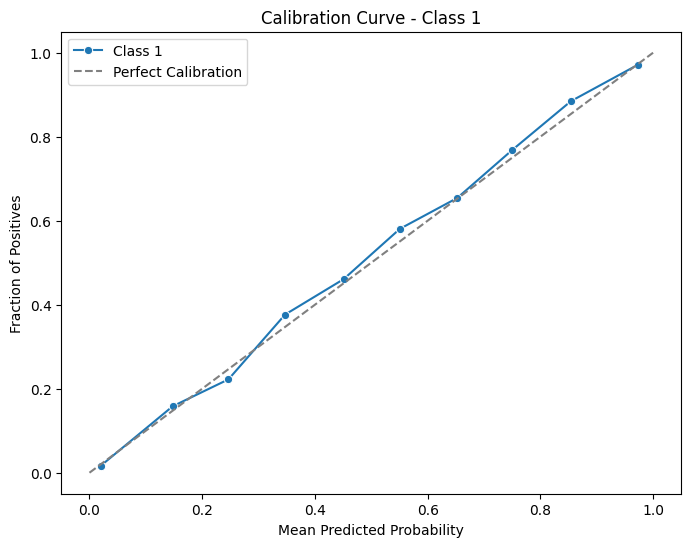


***Supplementary Figure S3.*** *Calibration curve for the Deletion class predictions*


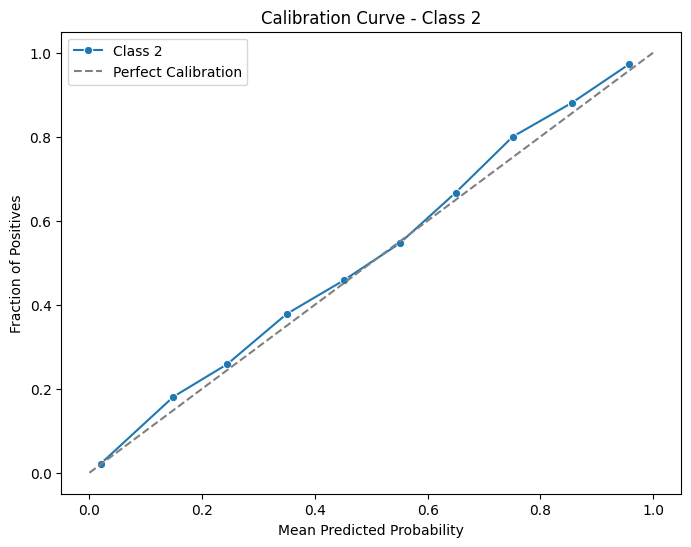


***Supplementary Figure S4.*** *Calibration curve for the Duplication class predictions*

***Supplementary Table S3.*** *Published ECOLE benchmarking summaries for established WES CNV callers, with CNN-Att results shown for contextual comparison. These results are not derived from a paired per-exon evaluation on the same 50-sample test set.*

| **Tool** | **DEL Prec** | **DUP Prec** | **NC Prec** | **DEL Recall** | **DUP Recall** | **NC Recall** | **DEL F1** | **DUP F1** | **NC F1** | **Overall F1** |
| --- | --- | --- | --- | --- | --- | --- | --- | --- | --- | --- |
| **XHMM** | 0.90 | 0.88 | 0.69 | 0.16 | 0.19 | 1.00 | 0.27 | 0.32 | 0.82 | 0.63 |
| **Control-FREEC** | 0.11 | 0.22 | 0.56 | 0.58 | 0.05 | 0.31 | 0.19 | 0.09 | 0.40 | 0.30 |
| **ECOLE** | 0.99 | 0.99 | 0.81 | 0.55 | 0.60 | 1.00 | 0.71 | 0.75 | 0.90 | 0.84 |
| **CNN‑Att** | 0.67 | 0.69 | 0.92 | 0.79 | 0.77 | 0.85 | 0.73 | 0.73 | 0.89 | 0.83 |


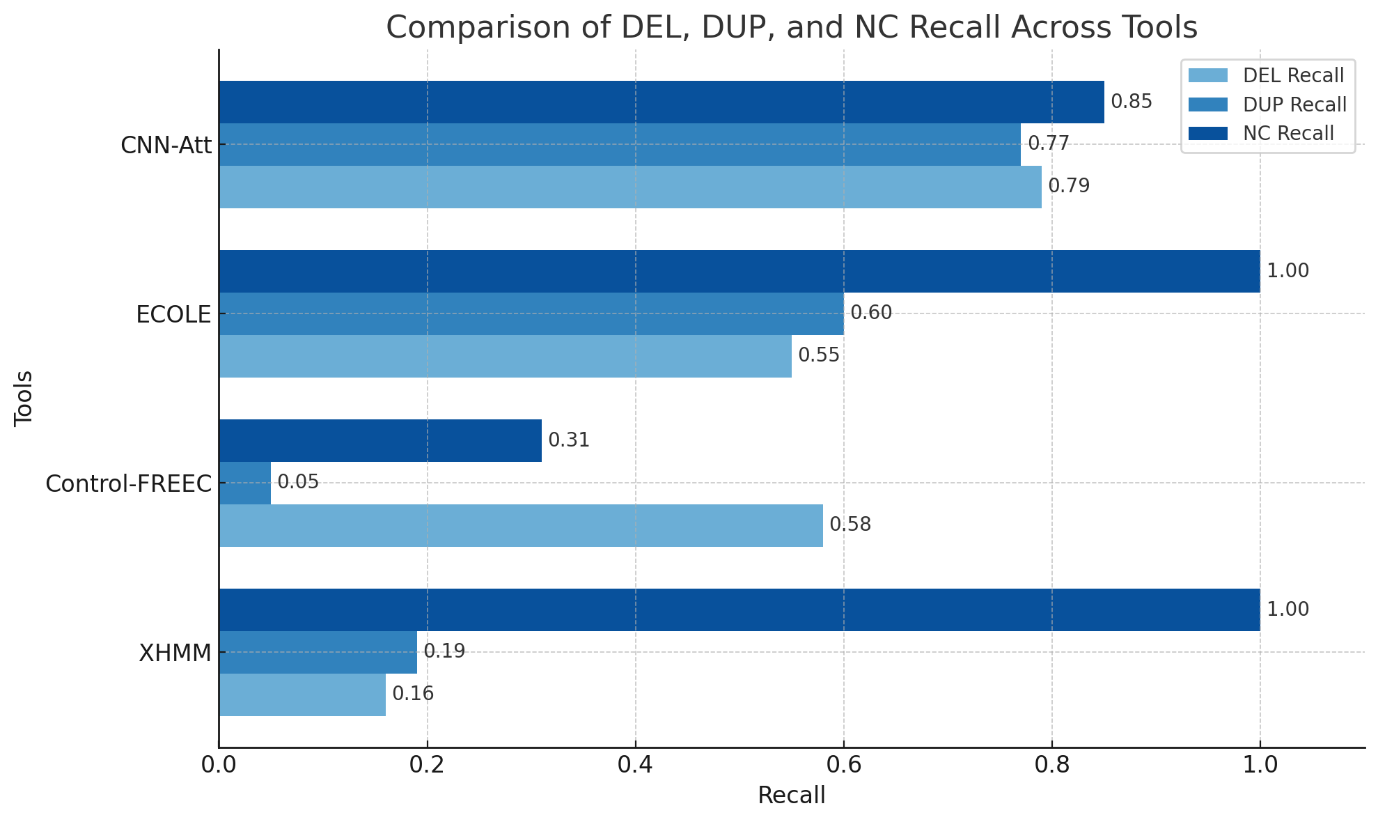


**Supplementary Figure S5***. Grouped bar chart showing per-class and overall recall for each tool.*

### **Cross-Platform Generalizability**

***Supplementary Table S4.*** *Detailed classification metrics for performance on WES data from HiSeq 4000, MGISEQ 2000, NovaSeq 6000, and BGISEQ 500*

|  |  | **DEL Precision** | **DUP Precision** | **NO CALL Precision** | **Overall Precision** | **DEL Recall** | **DUP Recall** | **NO CALL Recall** | **Overall Recall** | **DEL F1-score** | **DUP F1-score** | **NO CALL F1-score** | **Overall F1-score** |
| --- | --- | --- | --- | --- | --- | --- | --- | --- | --- | --- | --- | --- | --- |
| **HiSeq 4000** | XHMM | 0.00 | 0.00 | 0.45 | 0.20 | 1.00 | 0.00 | 0.00 | 0.45 | 0.00 | 0.00 | 0.62 | 0.28 |
|  | Codex2 | 0.22 | 0.50 | 0.45 | 0.41 | 0.19 | 0.23 | 0.66 | 0.41 | 0.20 | 0.31 | 0.53 | 0.39 |
|  | ECOLE | 0.96 | **0.98** | 0.71 | 0.86 | 0.50 | 0.77 | **1.00** | 0.81 | 0.66 | 0.86 | 0.83 | 0.80 |
|  | CNN‑Att | **0.98** | 0.85 | **0.88** | **0.90** | **0.83** | **0.91** | 0.91 | **0.89** | **0.90** | **0.88** | **0.90** | **0.89** |
| **MGISEQ 2000** | XHMM | 0.00 | 0.00 | 0.57 | 0.32 | 0.00 | 0.00 | 1.00 | 0.56 | 0.00 | 0.00 | 0.73 | 0.41 |
|  | CODEX2 | 0.14 | 0.16 | 0.51 | 0.35 | 0.14 | 0.08 | 0.63 | 0.40 | 0.14 | 0.10 | 0.57 | 0.37 |
|  | ECOLE | 0.88 | **1.00** | 0.73 | 0.82 | 0.40 | 0.57 | **1.00** | 0.78 | 0.55 | 0.72 | 0.84 | 0.76 |
|  | CNN‑Att | **0.94** | 0.91 | **0.99** | **0.96** | **0.97** | **0.96** | 0.95 | **0.96** | **0.96** | **0.93** | **0.97** | **0.96** |
| **NovaSeq 6000** | XHMM | 0.00 | 0.00 | 0.47 | 0.22 | 0.00 | 0.00 | 1.00 | 0.46 | 0.00 | 0.00 | 0.64 | 0.30 |
|  | CODEX2 | 0.51 | 0.21 | 0.49 | 0.44 | 0.21 | 0.25 | 0.69 | 0.44 | 0.21 | 0.33 | 0.59 | 0.42 |
|  | ECOLE | **1.00** | **1.00** | 0.64 | 0.83 | 0.43 | 0.58 | **1.00** | 0.74 | 0.60 | 0.73 | 0.78 | 0.73 |
|  | CNN‑Att | 0.85 | 0.93 | **0.91** | **0.90** | **0.81** | **0.88** | 0.96 | **0.90** | **0.83** | **0.90** | **0.93** | **0.90** |
| **BGI 500** | XHMM | 0.00 | 0.00 | 0.60 | 0.36 | 0.00 | 0.00 | **1.00** | 0.59 | 0.00 | 0.00 | 0.75 | 0.45 |
|  | CODEX2 | 0.85 | 0.91 | **0.98** | **0.94** | **0.97** | **0.98** | 0.91 | 0.94 | 0.00 | 0.06 | 0.60 | **0.94** |
|  | ECOLE | **0.93** | **0.97** | 0.78 | 0.85 | 0.48 | 0.63 | **0.99** | 0.82 | 0.64 | 0.77 | 0.87 | 0.81 |
|  | CNN‑Att | 0.85 | 0.92 | **0.98** | **0.94** | **0.97** | **0.98** | 0.92 | **0.94** | **0.90** | **0.95** | **0.95** | **0.94** |

### **Transfer Learning on Expert‑Curated Data**

***Supplementary Table S5.*** *Detailed classification metrics for* ***NA19240****.*

| **NA19240** | **DEL Precision** | **DUP Precision** | **NO CALL Precision** | **Overall Precision** | **DEL Recall** | **DUP Recall** | **NO CALL Recall** | **Overall Recall** | **DEL F1-score** | **DUP F1-score** | **NO CALL F1-score** | **Overall F1-score** |
| --- | --- | --- | --- | --- | --- | --- | --- | --- | --- | --- | --- | --- |
| ECOLE | 0.60 | 0.00 | 0.49 | 0.53 | 0.01 | 0.00 | 1.00 | 0.49 | 0.02 | 0.00 | 0.66 | 0.33 |
| **CNN‑Att** | **0.81** | **0.25** | **0.65** | **0.71** | **0.51** | **0.63** | 0.81 | **0.66** | **0.63** | **0.35** | **0.72** | **0.66** |

***Supplementary Table S6.*** *Detailed classification metrics for* ***HG00732****.*

| **HG00732** | **DEL Precision** | **DUP Precision** | **NO CALL Precision** | **Overall Precision** | **DEL Recall** | **DUP Recall** | **NO CALL Recall** | **Overall Recall** | **DEL F1-score** | **DUP F1-score** | **NO CALL F1-score** | **Overall F1-score** |
| --- | --- | --- | --- | --- | --- | --- | --- | --- | --- | --- | --- | --- |
| ECOLE | **0.83** | **0.40** | 0.61 | **0.67** | 0.03 | 0.06 | **0.99** | **0.61** | 0.05 | 0.10 | **0.76** | 0.48 |
| **CNN‑Att** | 0.60 | 0.21 | **0.71** | 0.64 | **0.38** | **0.58** | 0.72 | 0.60 | **0.46** | **0.31** | 0.72 | **0.61** |

***Supplementary Table S7.*** *Stratified performance of CNN-Att on true CNV windows only (DEL + DUP) in the 50-sample test set, grouped by training-set CNV frequency per exon window (fraction of occurrences of the same exon window in the 300-sample training matrix that were labeled DEL or DUP).*

| **Stratum** | **n_true_CNV** | **CNV_recall** | **CNV_F1** | **n_true_DEL** | **DEL_recall** | **DEL_F1** | **n_true_DUP** | **DUP_recall** | **DUP_F1** |
| --- | --- | --- | --- | --- | --- | --- | --- | --- | --- |
| **Never (0%)** | 6153 | 0.8815 | 0.9370 | 2961 | 0.8602 | 0.9248 | 3192 | 0.6388 | 0.7796 |
| **Often**  **(5–50%)** | 120 | 0.6500 | 0.7879 | 20 | 0.2500 | 0.4000 | 100 | 0.5700 | 0.7261 |
| **Majority (>50%)** | 30798 | 0.9364 | 0.9671 | 10175 | 0.8247 | 0.9039 | 20623 | 0.8360 | 0.9107 |

- CNV_recall is computed on true CNV windows only (DEL+DUP), as the fraction predicted as CNV (DEL or DUP).
- CNV_F1 is computed on the true-CNV-only subset using the binary prediction “CNV vs not-CNV”; because this subset contains no true negatives, this F1 is primarily driven by recall and should be interpreted as a complementary summary rather than a full CNV-vs-no-call F1 over all windows.
- Stratification is based on the fraction of occurrences of the same exon window (locus) in the 300-sample training matrix that were labeled as CNV (DEL or DUP).
- The “Often (5–50%)” stratum contains relatively few CNV windows and should be interpreted cautiously.
- No true CNV windows in the held-out test set fell into the Rare (0–5%) stratum.
